# PROTOCOL: Effectiveness of parent‐engagement programs to reduce truancy and juvenile delinquency: A systematic review

**DOI:** 10.1002/cl2.1189

**Published:** 2021-08-04

**Authors:** Sesha Kethineni, Susan Frazier‐Kouassi, Yuki Shigemoto, Wesley Jennings, Stephanie M. Cardwell, Alex R. Piquero, Kimberly Gay, Dayanand Sundaravadivelu

**Affiliations:** ^1^ Department of Justice Studies Prairie View A&M University Prairie View Texas USA; ^2^ Texas Juvenile Crime Prevention Center, College of Juvenile Justice & Psychology Prairie View A&M University Prairie View Texas USA; ^3^ Department of Psychology Prairie View A&M University Prairie View Texas; ^4^ Department of Criminal Justice and Legal Studies The University of Mississippi University Mississippi USA; ^5^ The University of Texas at San Antonio San Antonio Texas USA; ^6^ University of Miami Coral Gabes Florida USA; ^7^ John B. Coleman Library Prairie View A&M University Prairie View Texas USA

## Abstract

This review aims to synthesize the evaluation evidence for parent‐engagement programs that focus on reducing juvenile truancy as the primary outcome. Delinquent behavior will be assessed as a secondary outcome when included. This objective is guided by the following research questions: (1) what is the effectiveness of parent‐engagement programs for children in preschool (ages 4–5) through secondary education (ages 13–19) on primarily (a) reducing student truancy (i.e., unexcused or unauthorized absence) and secondarily (and when included) (b) reducing delinquent behaviors? (2) Is there variability in the effectiveness of parent‐engagement programs across moderators such as gender, age, grade levels, settings, and contexts? (3) What factors (e.g., groups, settings, and contexts) explain the variability in the effectiveness of engagement programs in a multivariate framework?

## 1 BACKGROUND

### 1.1 Description of the problem

Truancy is a global problem (Coughlan, 2009; Royal, 2015; UNICEF, 2016), yet there is no uniform definition of truancy. The common indicators include intentional absence from school without an excuse from parents or other authorities (Barry et al., 2011; Sinha, 2007). Also, there are different levels of truancy, depending on the number of unexcused absences. For example, a habitually truant student is someone who has a specific number of consecutive unexcused absences. In contrast, a chronic truant has already been disciplined for habitual truancy but continues to accumulate unexcused absences despite a court order or school mandate (Seeley, 2006).

There are also variations in the prevalence of truancy. In Scotland, for example, about 3% of students had unexcused absences in the 2016–2017 school year (Scottish Government, 2017). In the United States, truancy rates ranged from 10.8% to 11.1% between 2002 and 2014, respectively (Maynard et al., 2017). A Global School‐Based Student Health Survey of 33 countries reported that one out of three adolescents between the ages of 13 and 17 had been truant in the previous 30 days. The Bahamas and Uruguay had a truancy rate of 20%, whereas Kuwait, Oman, and Tokelau reported over 40% (Global Education Monitoring Report Team, 2017/2018).

Studies indicate that truancy has short‐term and long‐term negative implications (Henry, 2007; Mueller & Giacomazzi, 2006). For example, in the short term, truancy can result in poor academic performance, school dropouts, teen pregnancies, substance abuse, and delinquent behaviors (Henry, 2007, 2010; Henry & Huzinga, 2007; Henry & Thornberry, 2010; Rivers, 2010). In the long term, truancy could lead to adult violence and incarceration (Henry, 2007, 2010). Research has shown that truant youths are not a homogenous group and differ on key individual, school, family, community, and contextual risk factors (Darmody et al., 2008; Kearney, 2008; Maynard et al., 2012). Individual risk behaviors of truancy include dropping out of school; delinquent behaviors such as substance abuse, gang activity (Best et al., 2006; Henry & Thornberry, 2010; Henry, 2010; Shute & Cooper, 2015), bullying (Gastic, 2008); and future incarcerations (Baker et al., 2001; Monahan et al., 2014; Vaughn et al., 2013). School dropout rates increase the likelihood of criminal activity and add to the pipeline to prison. According to the U.S. Bureau of Justice Statistics, 75% of state prison inmates and 59% of federal inmates are high‐school dropouts (Harlow, 2003).

School risk factors for truancy include school culture, curriculum, poor teaching, negative school environment, poor relationships with teachers, dissatisfaction with school, and school disciplinary practices (Malcolm et al., 2003). Community and contextual risk factors include delinquent peer involvement, employment opportunities, neighborhood characteristics, levels of social support, community norms, and community violence (Henry & Huzinga, 2007; Lyon & Cotler, 2007; MacDonald & Marsh, 2004). Family‐related contributors include poverty, family conflict, parental education, parental attitude toward education, and involvement in their children's school (Malcolm et al., 2003; Romero & Lee, 2008).

Due to concern over the dwindling role of families in their children's learning and the resulting burden placed on schools, policymakers have recommended different strategies in educational policies, such as parent participation in their children's education and making parents accountable for their children's school attendance and behavioral issues (Avvisati et al., 2014; Henderson & Berla, 1994; Henderson & Mapp, 2002; Ishak & Fin, 2013). Countries like the United Kingdom, Australia, South Africa, Singapore, Bahamas, Malaysia, and some states in the United States classify truancy as an offense. They use truancy laws and legal contracts as a way to sanction parents for their children's nonattendance and behaviors (Global Education Monitoring Report Team, 2017/2018). Countries in Latin America use economic incentives for families to ensure their children attend school (Global Education Monitoring Report Team, 2017/2018). However, interventions that focused solely on institutional measures were ineffective in reducing absenteeism and improving school–legal guardian relationships (Atkinson, 2016; Avvisati, 2010).

Many countries have included parent engagement as part of their educational policy because the school‐parent coordinated approach plays a critical role in improving parent‐child communication, students' self‐efficacy about education, and reducing the risk of delinquent behaviors (Lau et al., 2012; Mandarakas, 2014; Smit et al., 2008; Vellymalay, 2012; Zhao & Akiba, 2009). However, there are factors outside of the formal educational setting that could contribute to children leaving school. In Africa and Asia, poverty, single‐parent households, parents' education, and employment status are believed to be reasons for leaving school (Momo et al., 2018).

### 1.2 The intervention

Given the multiple factors that contribute to truancy, prevention and intervention programs have focused on integrating school, community, family, law enforcement, and/or courts to best address this problem (Dembo & Gulledge, 2009). Interventions related to truancy vary and target different age groups, genders, risk factors, settings, and delivery of the intervention through various modalities. Although some programs focus on sanctions for truancy, recent interventions increasingly emphasize prevention through coordination of community, schools, families, and agencies (Sutphen et al., 2010).

This systematic review will focus on students in preschool through secondary education, with a primary goal of reducing truancy through parent engagement/involvement programs. A court may recommend the participation of parents, the school may recommend, or parents may participate voluntarily.

The review will include the interventions of varying dosages to examine the influence of lengths of parental intervention programs in reducing truancy. The curriculum may consist of information or workshops on child and adolescent development, problem‐solving, interpersonal communication, parenting style, support for a child's education, and connecting students and families to community resources. Interventions may be delivered by the class teacher, other school personnel, or nonschool personnel, such as community‐based organizations or by the court/probation department.

### 1.3 How the intervention might work

Interventions related to parental engagement in schools vary in terms of scope and method of delivery. The approaches used to address and engage parents in combating truancy range from a punitive approach (e.g., school or truancy court order) to a more supportive one where schools work in partnership with parents to encourage new and empowering behavior. Researchers increasingly recognize that parents and guardians tend to exert more control over factors that affect attendance than previously thought. In particular, in early grades (preschool through primary education), parents have the most influence over school routines that involve attendance (Robinson et al., 2018). Seldom do we find interventions with a logic or pathway model that outlines how the intervention will facilitate the desired outcomes. Likewise, although there is a lot of evaluation research on truancy, hardly any research meets the empirical requirements for combining and comparing multiple scientific studies (Keppens & Spruyt, 2020).

There are individual theories that touch on different aspects of parent involvement. Social Development Theory supports the notion that parental involvement is an important factor in a child's achievement (Tekin, 2011). Parental monitoring, parental acknowledgment of child's disclosure, and psychological control of the child are risk factors for delinquency (Hoeve et al., 2009). Ecological Systems Theory, proposed by Bronfenbrenner (1979), shows the impact of social, political, biological, and ecological factors on a child's development. In this review, we include parent engagement programs that address truancy and delinquency that incorporate the three domains: parent, child, and school factors. The proposed pathway model is based on an integrated theoretical framework of Social Development Theory, Ecological Systems Theory, and prior research.

The proposed pathway model helps us understand the process by which parents' involvement in a targeted program can reduce truancy and prevent delinquency in their children. The input factors include characteristics of (1) individual/parent, (2) children, and (3) program intervention, all of which contribute to parents' motivation to change. Individual characteristics refer to those characteristics that help determine the parents' capacity for change, including support for their child's education, parenting style, mental/physical health, and/or the presence of substance use/abuse. In addition, economic factors (McCluskey et al., 2004), including the parents' socioeconomic status, perceived costs and benefits, marital status, employment, and availability of childcare and/or transportation, may individually or collectively influence the parents' initial or continuing enrollment in the program. Family dynamics and characteristics have also been identified as contributors and/or barriers to school truancy for many students. Once parents are motivated to change, they are more likely to enroll in the program. A study of 10 school districts found that by supporting families, one could leverage families to improve student outcomes by successfully targeting parental beliefs and reducing student absenteeism (Epstein & Sheldon, 2002). Empowering parents as partners in their children's education has been found to positively affect student outcomes (Henderson & Mapp, 2002). However, several behavioral barriers, including limited attention, unsubstantiated beliefs, and low literacy, can affect parents' ability to process and act upon the information they receive about their child's education, thus creating a challenge for effective parent engagement (Damgaard & Nielsen, 2018). Intervention to change parents' mistaken beliefs about their children's attendance behaviors has been found to improve school attendance (Lasky‐Fink et al., 2021). Likewise, parents' perception of school legitimacy may hinder their motivation to participate in truancy intervention (Antrobus et al., 2019). If parents view that school authorities have failed to engage parents in procedural justice, they may not believe in the legitimacy of the authorities (Antrobus et al., 2019). Child characteristics include attitudes toward school and low academic performance, which may work together to increase the chances of truancy/delinquency. If a child fails in his/her schoolwork, it will affect his/her attitudes toward school, leaning toward negative perceptions of school legitimacy and attitudes, which then increases the chances of truancy. The characteristics of intervention (e.g., time and scheduling, whether court‐ordered vs. school‐sponsored, the duration of the program, the curriculum) will influence whether parents will decide to participate.

Each of the factors mentioned above is believed to contribute to parents' motivation to change their behavior and subsequently enroll in a truancy program. In addition, we expect an increase in parental engagement and/or improved parenting skills due to participation in the program. This increased parental engagement will contribute to a reduction in truancy and delinquency.

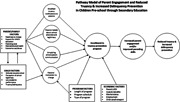

### 1.4 Why it is important to do this review

#### 1.4.1 Extant and ongoing reviews

The importance of partnerships between parents and teachers has been well‐documented over the last three decades (Fan & Chen, 2001; Lima & Kuusisto, 2020). However, extant reviews do not focus on parental engagement programs and their impact on truancy or used small samples or limited geographical regions. A search of Campbell and Cochrane's systematic review library was conducted to identify completed and ongoing reviews on the subject. The search revealed several reviews related to student behaviors such as aggression (Wilson & Lipey, 2006), bullying and victimization (Farrington & Ttofi, 2009), school disciplinary exclusion (Valdebenito et al., 2018), delinquent behaviors (Piquero et al., 2008), and school dropouts (Wilson et al., 2011). There was one systematic review that is relevant to this study, conducted by Maynard et al. (2013). The systematic review focused on student‐targeted and parent/family‐targeted interventions intended to reduce chronic truancy. However, the authors note small sample sizes and variations between studies as some of the limitations.

A few reviews focus on (1) the effect of parental involvement on children's behavioral outcomes (e.g., Cobb, 2009; Epstein & Sheldon, 2002; Hahn et al., 2007; McCarter et al., 2019; McNeal, 1999); (2) dropout programs on school completion and school dropout aimed at primary and secondary students (Wilson et al., 2011); (3) psychosocial interventions on reducing school anxiety and school attendance (Maynard et al., 2015); and (4) school‐wide positive behavior intervention and support program on school‐wide attendance (Berg, 2018). While there is a wide range of reviews of school intervention programs, none focus specifically on parental engagement programs as a mechanism to reduce truancy for different age groups, settings, and delivery methods.

#### 1.4.2 The potential application of review findings

Multiple countries have emphasized the importance of truancy reduction through demonstration programs (Barnsdale & Walker, 2007; Catalano et al., 1998; D'Aulerio et al., 2012) or other policies and legislation (Abu Dhabi Education Council, 2014–2015) For example, the WE‐STAY program has been implemented in six European counties to prevent truancy among adolescents through school‐based intervention programs that incorporate parent–school communications (D'Aulerio et al., 2012). In the Emirate of Abu Dhabi, all private schools require a contract between parents and schools in limiting school absences as well as involve parents/guardians in the learning and decision‐making process (Abu Dhabi Education Council, 2014–2015). In the United States, the truancy reduction demonstration program (1998) and the “No Child Left Behind Act” in 2001 paved the way for increasing attention to parent engagement in schools (Catalano et al., 1998; Lavery, 2016). The purpose of the proposed systematic review is to evaluate existing parent‐engagement programs that focus on reducing truancy. The review will examine various parent‐engagement programs, the curriculum, and outcomes across students from preschool (ages 4–5), primary/elementary (ages 6–12), and secondary education (ages 13–19); (2) provide empirical data on best practices; and (3) identify possible reasons for the success or failure of these programs. This systematic review of parent‐engagement programs will help inform policymakers and stakeholders on effective parent‐engagement programs in developing policies to address truancy. In particular, the review will help explain variations in practices to determine the impact of parent‐engagement programs across groups (gender, age, and grade levels), settings (school‐based vs. community), and contexts (voluntary participation vs. court mandated). The results will inform different audiences—researchers, policymakers, educators, and juvenile justice professionals—on the impact of parent‐engagement programs in reducing truancy.

## 2 OBJECTIVES

This review aims to synthesize the evaluation evidence for parent‐engagement programs that focus on reducing juvenile truancy as the primary outcome. Delinquent behavior will be assessed as a secondary outcome when included. This objective is guided by the following research questions:

1. What is the effectiveness of parent‐engagement programs for children in preschool (ages 4–5) through secondary education (ages 13–19) on primarily (a) reducing student truancy (i.e., unexcused or unauthorized absence) and secondarily (and when included) (b) reducing delinquent behaviors?
2. Is there variability in the effectiveness of parent‐engagement programs across moderators such as gender, age, grade levels, settings, and contexts?
3. What factors (e.g., groups, settings, and contexts) explain the variability in the effectiveness of engagement programs in a multivariate framework?

## 3 METHODS

### 3.1 Criteria for including and excluding studies

#### 3.1.1 Types of studies

The systematic review will include randomized control designs (including cluster‐randomized designs) and quasi‐experimental designs with a matched comparison group. Although quasi‐experimental designs tend to offer less compelling support for causal inferences, careful matching of subjects on key variables minimizes any issues with nonrandomization. If the study is eligible on all inclusion criteria and has mixed methods, it will be included in the review. If the study included is only qualitative data, it will be excluded. In addition, studies will be included regardless of publication status or date of publication.

#### 3.1.2 Nature of eligible comparison conditions

Eligible comparison conditions are placebo, no treatment, waitlist control, treatment‐as‐usual and alternative treatment.

#### 3.1.3 Types of participants

Studies that involve the parent(s) or legal guardian(s) who have participated in parent‐engagement/involvement programs will be included. The interventions should target children in early childhood education (ages 4–5), primary education (ages 6–12), and secondary education (ages 13–19). Some countries use additional classification such as middle‐school/junior high (ages 11–16) and high‐school (ages 17–19) identified as truant or having attendance problems (UNESCO Institute for Statistics ISCED, 2011). Studies that include diverse populations regarding race, ethnicity, gender, socioeconomic status, and geography will be included from both published and unpublished sources. Studies that target children who are physically and mentally disabled will be excluded. Parents of children with developmental disabilities and/or special needs experience different challenges compared to parents of “typically developing children” (Woodman, 2014). Often, schools provide Individualized Education Plan and parental engagement programs geared toward children with developmental disabilities and/or special needs, which are different than what schools typically provide for truancy (DuPaul et al., 2018).

#### 3.1.4 Types of interventions

A parent engagement program for reducing truancy behavior may be characterized by parents/guardians obtaining knowledge through informational resources and or participation in activities or seminars that are geared toward identifying specific barriers to their children's school attendance. The goal of these programs is to enable the parents/guardians to actively improve school attendance for their children using skills learned in the program. For example, a parent engagement program may consist of information or workshops on child and adolescent development, problem‐solving, interpersonal communication, parenting style, support for a child's education, and connecting students and families to community resources. There are variations in truancy intervention programs (TIPs) by setting. Still, the essential focus of the intervention is to involve parents in truancy prevention. They are often grouped by setting: school‐based, community‐based, school and community‐based, court‐based, school‐law enforcement‐based, and programs offered in other settings (Dembo & Gulledge, 2009). For example, the Check and Connect (C&C) program is a school‐based truancy intervention developed in the United States and offered in other countries, includes individual and family needs such as family communication, problem‐solving, and tutoring (Guryan et al., 2020; Janosz et al., 2019). Interventions such as Multisystemic Therapy targets environmental risk factors addressing problem behaviors of youth at home, school, and community (Littell et al., 2005). Court‐based programs such as Truancy Court Diversion Program is a voluntary program where parents and students at risk of chronic truancy participate in group and individual sessions (Cahill & Liberman, 2012). The Ability School Engagement Program is a collaborative program with youth, parents, and police tested in Australia (Mazerolle et al., 2019). Others use a multidisciplinary approach. For example, the TIP works with youth, parents, teachers, social work staff, and community members. The programs hold parents accountable, and at the same time, ensure that students are given education, assessment, and services (Smink & Reimer, 2005). Interventions such as Partnering to Assess and Counteract Truancy work with students, families, and community members. Another court and/or community‐based program, the Positive Steps program, consists of case management, parent mentoring, Functional Family Therapy, and multidisciplinary teams (Washington State Institute for Public Policy, 2009).

#### 3.1.5 Types of outcome measures

Truancy (unexcused or unauthorized absences) is the primary outcome of interest in this review. The studies must include at least one quantifiable measure of unauthorized or unexcused school absenteeism, which may be assessed by a scaled measure or an exact number of absences. A repeated number of unauthorized or unexcused absences within a specific timeframe is considered truancy. The secondary outcome of delinquent behavior (i.e., substance abuse, gang activity, and aggression) will be also be included. Studies that focus solely on outcomes other than truancy, such as academic achievement, educational attainment, and mental and/or psychological issues, will be excluded. The review will include interventions that collect data on school absenteeism in four ways: self‐report surveys from youth, official data (from school, courts, police, or probation), or reports from parents and teachers, and other sources (i.e., professional observation, assessment, or diagnosis).

#### 3.1.6 Duration of follow‐up

The duration of follow‐up varies from the start of the intervention to the treatment completion in terms of mean time (i.e., day or weeks) among studies to be reviewed. There are interventions that measure the level of parental involvement at a single point in time and also over time (Blondal & Adalbjarnardottir, 2009; Skaliotis, 2009). Regardless of variations in the timeframe of follow‐up, the review will include both short‐term and longitudinal studies. The synthesis of different follow‐up periods is detailed in the Section 3.3.7.

#### 3.1.7 Types of settings

The review will include international studies conducted in both urban and rural settings. Also, the review will include interventions delivered by school, community, probation, law enforcement, or court. Finally, the review will include studies conducted in residential facilities (e.g., juvenile institutions and treatment facilities) and psychiatric institutions if they meet the eligibility criteria.

### 3.2 Search methods for identification of studies

The research on the link between parental engagement and truancy is a topic that crosses multiple disciplines (e.g., criminology, sociology, psychology, education, legal studies, social work, and public health). Thus, a comprehensive search of multiple electronic databases, academic journals, research registries, and gray literature will be conducted. The exact search terms and search locations are described in the following subsections.

#### 3.2.1 Search strategy

Search strings will be created utilizing Boolean operators “AND” and “OR” to combine search terms across participant, outcome, and evaluation categories (see Table 1). Searches will be conducted across the title, abstract, keywords, and subject/indexing term fields in databases, but will be adapted if these fields are not available.

**Table 1:** Search terms and structure

Step	Search fields	Search syntax
1	Title	truan* OR “unauthorized absen*” OR “unauthorised absen*” OR “unexcused absen*” OR absent OR “skip* school” OR absenteeism OR “school attendance” OR “school dropout*” OR “school drop‐out*” OR “school drop out*” OR “school refusal”
2	Abstract	truan* OR “unauthorized absen*” OR “unauthorised absen*” OR absent OR “unexcused absen*” OR “skip* school” OR absenteeism OR “school attendance” OR “school dropout*” OR “school drop‐out*” OR “school drop out*” “school refusal”
3	Keywords	truan* OR “unauthorized absen*” OR “unauthorised absen*” OR “unexcused absen*” OR absent OR “skip* school” OR absenteeism OR “school attendance” OR “school dropout*” OR “school drop‐out*” OR “school drop out*” “school refusal”
4	Subject or Indexing Terms	truan* OR “unauthorized absen*” OR “unauthorised absen*” OR “unexcused absen*” OR “skip* school” OR absenteeism OR “school attendance” OR “school dropout*” OR “school drop‐out*” OR “school drop out*” “school refusal”
5	‐	1 OR 2 OR 3 OR 4
6	Title	parent* OR caregiver* OR family OR families OR “care giver*” OR care‐giver*
7	Abstract	parent* OR caregiver* OR family OR families OR “care giver*” OR care‐giver*
8	Keywords	parent* OR caregiver* OR family OR families OR “care giver*” OR care‐giver*
9	Subject or Indexing Terms	parent* OR caregiver* OR family OR families OR “care giver*” OR care‐giver*
10	‐	6 OR 7 OR 8 OR 9
11	Title	evaluat* OR experiment* OR interven* OR program* OR random* OR RCT OR treat* OR trial*
12	Abstract	evaluat* OR experiment* OR interven* OR program* OR random* OR RCT OR treat* OR trial*
13	Keywords	evaluat* OR experiment* OR interven* OR program* OR random* OR RCT OR treat* OR trial*
14	Subject or Indexing Terms	evaluat* OR experiment* OR interven* OR program* OR random* OR RCT OR treat* OR trial*
15	‐	11 OR 12 OR 13 OR 14
16	‐	5 AND 10 AND 15

*Note:* Exact syntax and wildcards will be adjusted for each database and reported in the final review.

Eligible studies will not be restricted to any written language or date of publication. For non‐English publications, first, we will use Google Translate for title and abstract screening. Once the eligible studies are identified, we will translate the main text using Google Translate for full‐text review. Finally, if we find the missing data, we will attempt to contact the study authors for missing information.

#### 3.2.2 Electronic searches

An extensive search of various databases and gray literature sources relevant to the topic will be conducted, as outlined in Table 2.

**Table 2:** Search location for the programs to reduce truancy

Indexed & academic databases	
ProQuest	American Periodicals Criminal Justice Database EBook Central Music Periodicals Database PAIS Index Periodicals Archive Online ProQuest Dissertations & Theses Global Publicly Available Content Database Research Library Social Science Database
EBSCOhost Research Databases	Academic Search APA PsycArticles APA PsycInfo Criminal Justice Abstracts with Full Text Educational Administration Abstracts ERIC Psychology and Behavioral Sciences Collection Social Science Full Text
Standalone Databases	Campbell Systematic Reviews journal Cochrane Library o Cochrane ‐ Database of Systematic Reviews o Cochrane Reviews o Cochrane Protocols Cochrane Central Register of Controlled Trials (CENTRAL) National Criminal Justice Reference Service (NCJRS) The International Prospective Register of Systematic Reviews (PROSPERO; https://www.crd.york.ac.uk/prospero/) The Evidence for Policy and Practice Information and Co‐Ordinating Centre (EPPI‐Centre; http://eppi.ioe.ac.uk/cms/)
Grey Literature Sources & Websites	Collaborative for Academic, Social, and Emotional Learning (CASEL; https://casel.org/) Evidence‐Based Program Database (http://www.alted-mh.org/ebpd/) Google Scholar, using the search terms OpenGrey OECD iLibrary Institute for Research on Higher Education. (http://www.ed.gov/about/offices/list/ies/index.html) National Center for Public Policy and Higher Education. (http://www.highereducation.org/) National Registry of Evidence‐Based Programs and Practices World Health Organization (WHO) WorldCat
Searches of Academic Journals	*Journals in Education (Scimago Journal & Country Rank ‐‐* https://www.scimagojr.com/journalrank.php?category=3304%26order=h%26ord=desc%26page=1%26total_size=1401 *)* *Africa Education Review* *American Educational Research Journal* *American Journal of Education* *Asia Pacific Journal of Education* *Australian Journal of Education* *British Educational Research Journal* *British Journal of Educational Psychology* *British Journal of Sociology of Education* *Children and Youth Services Review* *Educational Evaluation and Policy Analysis* *European Journal of Education* *European Journal of Education & Psychology* *Journal of Education Psychology* *Journal of School Psychology* *Journal of Youth and Adolescence* *Review of Educational Research* *School Psychology* *School Psychology Review* *Social Science Research* *Sociology of Education* *British Journal of Criminology* *Child and Youth Services Review* *Crime and Delinquency* *Criminology* *Education and Urban Society* *International Journal of Child, Youth, Family Studies* *Journal of Child and Family Studies* *Journal of Experimental Criminology* *Journal of Youth Studies* *Social Forces*

### 3.3 Data collection and analysis

#### 3.3.1 Description of methods used in primary research

An example representing methods for anticipated studies includes an RCT conducted in France by Avvisati et al. (2014). Avvisati et al.'s (2014) study of parents' involvement in the education of middle‐school children in deprived schools in France included 34 schools that volunteered for the study. The study selected 183 classes and parents of about 4300 6th graders to participate in parent‐school meetings on how to make parents better involved in their childrens' education. First, the researchers randomized classes within each school, resulting in 96 classes in the treatment and 87 classes in the control group. Further randomization resulted in four groups within each school: volunteers in test classes, nonvolunteers in test classes, volunteers in control classes, and nonvolunteers in control classes. The outcomes included: (1) “parental involvement attitudes and behaviors; (2) children's noncognitive skills, as reflected by their disciplinary record and work effort; and (3) children's cognitive achievement, such as academic results” (p. 15). The program included three sessions of 2 hours each, and the outcomes were measured at the end of the school year (including truancy and discipline) and an 18‐month follow‐up. An example of a quasi‐experimental study was conducted by McNeely et al. (2019) in the United States. The researchers evaluated the TIP in improving school attendance of students in grades 7–10 who were referred to court. The program includes school staff, parents, and the juvenile justice system. The study utilized a multilevel matching process to select the experimental group and comparison group of public‐school students who are eligible to participate in a court‐ordered TIP. The experimental group (*n* = 4412) consisted of students grades 7th through 10th referred to TIP. The comparison group (*n* = 9111) consisted of TIP eligible but not referred to the program. The primary outcome measure was the students' annual daily attendance rate, measured as the proportion of days enrolled in any public school in the state. The secondary outcome measure used was the number of excused and unexcused absences in the month following referral to TIP.

#### 3.3.2 Criteria for determination of independent findings

Because studies may report outcomes based on the same data set in multiple articles, we will follow the detection heuristic proposed by Wood (2008) for inspecting duplicate results (e.g., ”Does the study share 1 or more authors with another study?” and “Are study characteristics sufficiently different to exclude the study?”). When studies are determined to be using the same data set and resulting in two or more publications, these publications will be coded individually, but they will be treated as a single study. In addition, if the samples used in multiple studies are considered as partly overlapping (e.g., researchers published an article using preliminary data and then published another article using the complete data), the study with the largest sample size reported will be included in the meta‐analysis. However, when studies include independent samples, we will use the relevant primary outcome of truancy (and, secondarily, delinquency when included), and we will estimate the effect sizes as long as the studies met the inclusion criteria mentioned earlier. As the primary outcome (i.e., truancy) and the secondary outcome (i.e., delinquency) are conceptually distinct, the effect sizes will be generated separately for these outcomes.

#### 3.3.3 Selection of studies

If the intervention has the stated goal of reducing truancy and has parent engagement or participation as the primary aim of the research, they will be checked for eligibility.

A team of trained doctoral research assistants will upload all abstracts and titles identified by the search into RefWorks. Once the duplicate entries are removed, the primary coders/screeners will independently screen abstracts using the following screening criteria (see Appendix A):

a. Does the study mention parent/family engagement/intervention/involvement? Yes/No
b. Does the study address truancy/school dropouts/school absenteeism? Yes/No

If any of the screening eligibility criteria is not met, the documents will be classified as ineligible. Documents that meet the initial title/abstract eligibility criteria will move to the full‐text eligibility screening phase, whereby the full texts will be accessed and reviewed in DistillerSR program by the primary screeners via the data collection forms. Any disagreements of the full‐text screening will be reviewed by the research team (including the methodologists). The final eligibility screening will be done using the following eligibility screening criteria:

- Involves parent engagement/intervention program; AND
- Utilizes randomized or quasi‐experimental design; AND
- Involves parents of children who are in early childhood education (ages 4–5), primary education (ages 6–12), and secondary education (ages 13–19). Some countries use additional classification such as middle‐school/junior high (ages 11–16) and high‐school (ages 17–19); AND
- Program conducted in school, court, probation, juvenile institutions, psychiatric facilities, or community setting; AND
- Primary outcome measure is truancy.

The doctoral research assistants will receive training related to information retrieval, screening, coding, and storage of data for quality assurance. The reference librarian will assist the students in the search process. If electronic copies are not available, the reference librarian and the review team member will attempt to secure the print version via the Prairie View A&M Interlibrary Loan Office.

To ensure consistency in screening decisions, each primary screener will screen 30 full‐text documents for eligibility. Percentage agreement between the primary screeners for document eligibility will be calculated for inter‐rater reliability of 95% agreement. If inter‐rater reliability falls below the threshold of 95% percent, further training will be provided to the primary screeners. Finally, the discrepancy in coding among the coders will be resolved by a discussion between the coders and the lead researchers.

#### 3.3.4 Data extraction and management

Data will be extracted from all eligible studies using data collection forms in DistillerSR and hosted online (see Appendix A). The lead researchers will check the consistency of coding among the coders by implementing a similar quality control process using the initial eligibility screening. At least 10% of the sample of each coder will be double‐coded on methodological risk of bias and effect size to verify coding reliability. In addition, one of the lead researchers will resolve any discrepancies. The primary categories for coding will consist of:

- Reference information (i.e., type of publication, funding source, and publication title);
- Study context and sample characteristics (i.e., place where the study was conducted, target population, and who initiated the program, age, gender, and race/ethnicity);
- Intervention characteristics (i.e., length of intervention, type of intervention, and type of curriculum);
- Study design and methods (i.e., randomized or quasi‐experimental, primary and secondary outcomes, and type of data used); and
- Effect size and statistical significance (i.e., effect size, validity estimates, means and standard deviations, and significance tests).

#### 3.3.5 Assessment of risk of bias

In assessing the risk of bias among the studies included in the meta‐analysis, we will follow the Risk of Bias in Non‐randomized Studies of Intervention (ROBINS‐1; Sterne et al., 2016) for nonrandomized studies, such as quasi‐randomized studies, and also follow Version 2 of the Cochrane tool for assessing the risk of bias in randomized trials (RoB 2; Sterne et al., 2019) for randomized studies. In particular, when assessing the nonrandomized studies, seven bias domains will be assessed: confounding, selection of participants, classification of interventions, deviations from intended interventions, missing data, measurement of the outcomes, and selection of reported results (Sterne et al., 2016). Regarding the randomized trials, five bias domains will be assessed: randomization process, deviations from intended interventions, missing outcome data, measurement of the outcomes, and selection of the reported results (Sterne et al., 2019).

#### 3.3.6 Measures of treatment effects

Individual effect sizes will be taken directly from the articles, if reported. If effect sizes are not reported, statistics from the articles will be used to attempt to calculate an effect size. As we expect that outcome measures will be mostly continuous, standardized mean differences effect sizes (i.e., Cohen's *d*) will be used. When outcome measures are binary, the effect sizes will be calculated as odds ratios, and they will be transformed to standardized mean differences. Standard “rules of thumb” for Cohen's *d* will be used to evaluate the magnitude of the effect sizes.

#### 3.3.7 Unit of analysis issues

Educational intervention studies are often clustered (Walsh et al., 2018), such as when an intervention is administered to a group of participants by teachers or therapists. This clustering needs to be taken into account in meta‐analyses because it may overestimate the precision of the study, giving larger weight to the study (Higgins et al., 2020). As the clustering issue applies to studies where individuals are randomized, but the intervention is delivered in clusters and/or where clusters are not randomized (quasi‐experiments), a binary variable (yes/no) will be coded in the event the research team is able to identify that the author/s explicitly adjusted their analysis for clustering as described in the study or the ability for the research team to ascertain that cluster mean analysis, multilevel models, mixed regression, generalized estimated equations, or clustered robust standard errors were used. However, for those included studies that used clusters (randomized or quasi‐experiments) that did not adjust for clustering or when it was unable to determine if they had adjusted for clustering, the standard errors of the effect sizes will be adjusted using the approach suggested by Higgins et al. (2020).

Regarding crossover trials (i.e., all participants receive multiple interventions), it is anticipated that we will not locate studies using such a research design. However, if there is a study using crossover trials, mean differences will be obtained for use in the meta‐analysis using the approach suggested by Higgins et al. (2019).

In a situation where a study involves multiple time points, the outcomes will be recorded for each time point. Next, time frames will be categorized to reflect short‐term (i.e., immediately after the intervention to 3 months), medium‐term (i.e., 4–6 months after the intervention), and long‐term (i.e., 7 months and more following the intervention), follow‐up and separate analyses will be conducted for each category. However, as the reviewed studies may not equally distribute to these categories, the time frames may be adjusted after the recording of the studies are complete.

When there are studies with two or more treatment groups, the relevant intervention groups will be identified, and the other groups will be left out from further analyses. When multiple groups were determined as relevant, to avoid the issue of nonindependence, the researchers will combine these groups into a single group and conduct a single comparison (Higgins et al., 2020). Specifically, the mean of the combined group will be calculated by:

$$\frac{N_{1}M_{1}+N_{2}M_{2}}{N_{1}+N_{2}}.$$

Furthermore, the standard deviation of the combined group will be calculated by:

$$\sqrt{\frac{{(N}_{1}-1)\mathrm{SD}{}_{1}^{2}+{(N}_{2}-1)\mathrm{SD}{}_{2}^{2}+\frac{N_{1}N_{2}}{N_{1}+N_{2}}{(M}_{1}^{2}+M_{2}^{2}-{2M}_{1}M_{2})}{N_{1}+N_{2}-1}}.$$

#### 3.3.8 Dealing with missing data

In cases of missing data on effect sizes, the research team will contact the lead author for statistical information such as *t*, *F*, *p*, or *z* values to convert to effect sizes. If the researchers cannot provide the data, the study will be excluded from the meta‐analysis. However, we will include the review in the summary of the study and the risk of bias.

#### 3.3.9 Data synthesis

The R package metafor (Viechtbauer, 2010) will be used to conduct both overall meta‐analysis and subgroup analysis. A meta‐analysis will be conducted on examining the effect of parental‐engagement programs on the primary outcome of truancy (e.g., unexcused and/or unauthorized school absences) and secondarily delinquent behavior (when included). The independent variable will be the parental‐engagement program, and the primary dependent variable will be truancy with delinquency being the secondary outcome (when reported). In addition, the inverse‐variance weighted method of meta‐analysis will be used. For the random‐effects analysis, the between‐studies variance will be estimated using a restricted maximum likelihood (REML) method. The results of meta‐analyses will be presented using a forest plot with 95% confidence intervals.

#### 3.3.10 Subgroup analysis and assessment of heterogeneity

To assess for heterogeneity among the primary outcome variable (truancy) and for the secondary outcome variable, delinquency (when included), *Q* test and *I* ^2^ statistics (Higgins et al., 2003) will be conducted. When there is sufficient data, a random effects meta‐regression (i.e., mixed effects model) will be conducted to examine whether the primary and secondary outcome of the intervention changes depending on the characteristics of the intervention (i.e., length of intervention, type of intervention, and type of curriculum) and the sample (i.e., place where the study was conducted, target population, who initiated the program, age [5–11‐/12–18‐year‐old], gender, and race/ethnicity). Furthermore, the moderating effect of the classifications of risk of bias on the effect of parental‐engagement programs on truancy will also be examined. Additional subgroup analyses may be conducted based on other characteristics of the reviewed studies, and if that is the case, we will differentiate the results of subgroup analyses that were conducted a priori and post hoc. Considering that there have been no studies conducting power analysis for moderation models, as recommended, robust variance estimation will be used when examining the moderating effects (Pigott & Polanin, 2020).

#### 3.3.11 Sensitivity analysis

To increase the robustness of the results, sensitivity analyses will be conducted when encountered with each of the following conditions: (1) When during the search for studies, the results reported in an abstract cannot be confirmed in the publication; (2) The majority, but not all of the participants in a study meet the inclusion criteria; and (3) There are missing outcomes, and as a result, adjusted and unadjusted estimates of the effect will be compared. Additional sensitivity analyses may be conducted when studies that appear as an outlier are identified during the investigation. Specifically, when conducting sensitivity analyses, we will rerun the meta‐analysis without the specific subgroups and compare any changes on the overall effect.

#### 3.3.12 Publication selection bias

To assess for publication bias, we will examine the funnel plot and also estimate the effect sizes using the procedure by Duval and Tweedie (2000), which would account for the publication bias.

#### 3.3.13 Treatment of qualitative research

Qualitative research studies will not be included in the current review.

## ROLES AND RESPONSIBILITIES

- *Content*: The lead reviewer, Sesha Kethineni, has extensive experience in the area of juvenile justice and program evaluation. She has conducted research and coauthored a book, *Comparative Delinquency: India and the United States* (1996), as well as several articles in peer‐reviewed journals as well as conference presentations. She also works as a consultant with the juvenile justice/court services agencies; and juvenile justice councils that address issues about truancy and delinquency. Susan Frazier‐Kouassi has extensive experience in program management and working with interdisciplinary research teams. She has worked in grant proposal development, writing, and reviews. In her most recent role, she served as the director of training and community engagement. Sesha Kethineni and Susan Frazier‐Kouassi will take the lead in organizing the content area as well as managing the team.
- *Systematic review methods*: Sesha Kethineni and Susan Frazier‐Kouassi have extensive experience in conducting literature searches and reviews. They will share responsibility for developing the literature review and monitor the coding by doctoral students. Stephanie M. Cardwell will assist in the systematic review search process.
- *Statistical analysis*: Yuki Shigemoto has substantial experience with advanced statistical analysis, including ANOVA, regression, structural equation modeling, hierarchical linear modeling, Bayesian statistics, survival analysis, and meta‐analysis. He also worked as a graduate research assistant at the Institute for Measurement, Methodology, Analysis, and Policy (IMMAP) at Texas Tech University for 2 years under the guidance of Dr. Todd Little, who is a founding director of IMMAP. At IMMAP, he also assisted program evaluation for NSF and NIJ research grants and served as a statistical consultant during the Annual Texas Tech Summer Institutes in Statistics in 2015 and 2017. Yuki Shigemoto will take the lead in statistical analysis for the systematic review.
- Wesley G. Jennings has over 250 publications, his h‐index is 55 (i‐index of 158), and he has over 10,000 citations to his published work. He has been actively involved in a number of systematic reviews and meta‐analyses on a range of topics, including family/parent training, self‐control improvement programs, and domestic violence.
- Alex R. Piquero has published over 400 peer‐reviewed articles in the areas of criminal careers, crime prevention, criminological theory, and quantitative research methods and has collaborated on several books. His work has been cited over 44,000 times (h‐index = 113), and he has been ranked as the #1 criminologist in the world since 1996 in terms of scholarly publications in elite criminology/criminal justice journals. A 2019 article in *PLOS Biology* identified him as being included among the top 100,000 most‐cited scientists in the world. He has extensive experience in conducting systematic reviews.
- Stephanie M. Cardwell has been involved in the evaluation of the Ability School Engagement Program. Wesley Jennings, Stephanie M. Cardwell, and Alex R. Piquero will assist Yuki Shigemoto with the statistical analyses for the project. Alex R. Piquero will also assist in drafting parts of the final report.
- Information retrieval: Kimberly Gay is the head of the Reference and Information Services Department at the Prairie View A&M University library. She previously served as an academic reference and instruction librarian at the same institution. She has taught, developed, and organized library information literacy classes for the College of Juvenile Justice and Psychology. Kimberly Gay will provide leadership in information retrieval utilizing the databases of the library and other sources.
- Supervision of data coding: Dayanand Sundaravadivelu is a doctoral student in the Department of Justice Studies and works as a graduate assistant in the Texas Juvenile Crime Prevention Center at Prairie View A&M University. He will supervise the data coding and data entry by three doctoral students, Tinu Neha Miriyam, Regan Rochelle Reid, and Shantol McIntosh.

## SOURCES OF SUPPORT

No financial and other support for the proposed review will be requested.

## DECLARATIONS OF INTEREST

Stephanie M. Cardwell and Alex R. Piquero have been involved in the evaluation of the Ability School Engagement Program (ASEP) intervention, including peer‐reviewed publications evaluating the program. ASEP is a third‐party policing intervention designed to increase school attendance and reduce antisocial behavior and would therefore qualify for inclusion as an eligible intervention. Stephanie M. Cardwell is also a coprincipal investigator who has been involved in the development of an upscaled test of ASEP currently in progress. To minimize potential bias, other authors will screen and code any papers co‐authored by Stephanie M. Cardwell and Alex R. Piquero that may be eligible for the study. Per requirements, they will not be involved in data extraction, coding, or critical appraisal of any eligible studies that they authored or any papers that utilize ASEP data as part of the systematic review.

## PRELIMINARY TIMEFRAME

We anticipate the final submission of the systematic review on or before March 2022.

## PLANS FOR UPDATING THE REVIEW

We will attempt to update this review 3–4 years after publication, subject to availability of funding and research team availability.

## APPENDIX A ABSTRACT AND FULL‐TEXT CODING

**1a. [StudyID]**

**Study Identification Number: The Unit that will be used to code here applies to a single study. First, conduct the title and abstract search**.

If there are several reports of a single study, the coding should be done from a full set of relevant reports using the best report of each item coded (i.e., subject samples or subsamples, treatments, measures, and statistical analyses). Also, if a single report describes more than one study sample (e.g., an evaluation at three different sites), each study sample will be given a unique study identification number, and each study will be coded as a separate report. For example, StudyID = 105 and each report will have an identification number (ReportID = 105.01, 105.02, 105.03). The ReportID will have two parts: the number before the decimal is the StudyID and the number after the decimal is the ReportID.

**1b. [Report‐ID]**

**2a. [Prim_Coder 1]**

**Coder Initial: DS**

**2b. [Secondary Coder]‐**

**Coder Initials: RR VV**

**Lead Team SK SFK**

**TITLE AND ABSTRACT ELIGIBILITY CHECKLIST:**

The document must include (3a) parent engagement/intervention/involvement component and (4) primary outcome of truancy (school dropout, school absenteeism, unauthorized or unexcused absences, school attendance, skipping school, or school refusal).

**[Eligibility]**

3a. Related to parent (family or caregiver) engagement/intervention/involvement/_____________**Yes**/No

3b. Related to the primary outcome of truancy (school dropout/school absenteeism/unauthorized or unexcused absences, school attendance, skipping school, or school refusal)/______**Yes**/No

***IF THE ANSWER IS YES, SELECT THE TITLE AND ABSTRACT FOR INCLUSION***

**FULL‐TEXT ELIGIBILITY CHECKLIST**

***Inclusion Criteria***

A study must meet the following criteria to be eligible. **REPEAT ITEMS 3A and 3B. FOR FULL‐TEST REVIEW**, Parent (family or caregiver) engagement/involvement and truancy (school dropout, school absenteeism, unauthorized or unexcused absences, school attendance, skipping school, or school refusal) are the primary outcomes for the review. The studies must include (1) either randomized experimental designs or quasi‐experimental designs with matching experimental and comparison groups (eligible comparison conditions are placebo, no treatment, waitlist control, treatment‐as‐usual and alternative treatment); and (4) conducted in School, court, probation, juvenile institutions, psychiatric facilities, or community setting. All these criteria must be met for the study to be included in the review. Answer each question with a “yes” or a “no.”

**4a. [Inclusion‐1]**

Is the study evaluation of a parent (family or caregiver) engagement program/involvement/intervention aimed at reducing truancy (school dropout, school absenteeism, unauthorized or unexcused absences, school attendance, skipping School, or school refusal)?

1. Yes
2. No

**4b. [Inclusion‐2]**

Does the study involve a randomized experiment (RCT), quasi‐experimental (eligible comparison conditions are placebo, no treatment, waitlist control, treatment‐as‐usual and alternative treatment), or evaluation design?

1. Yes
2. No

**4c. [Inclusion‐3]**

Is the study conducted in school, court, probation, juvenile institutions, psychiatric facilities, or community settings**?**

1. Yes
2. No
3. Others ………………(Specify)

**4d. [Inclusion‐4]**

Does the study include a comparison or a control group that did not receive the treatment condition?

1. Yes
2. No

**4e. [Inclusion‐5]**

Does the study report primary outcome of truancy (school dropout, school absenteeism, unauthorized or unexcused absences, school attendance, skipping School, or school refusal)?

1. Yes
2. No

**4f. [Inclusion‐6]**

Does the program apply to students in:

1. Early childhood/preschool
2. Primary School
3. Middle School
4. Secondary/High School
5. Specific grade (if given)________________

**5a. [Eligibility Status]**

1. Eligible
2. Not eligible (no further coding needed)
3. Relevant review

**5b. [Relevance]**

If the study does not meet the criteria above, answer the following question:

Is the study a review article that is relevant to this project (e.g., may have references to other studies that are useful, may have pertinent background information)?

1. Yes
2. No

**DUPLICATE STUDY**

To detect fully or partially duplicate study samples, a detection heuristic was adapted from Wood (2008).

**6a. [Detection1]**

Does the study share 1 or more authors with another study?

1. Yes (proceed to Detection2)
2. No (skip to the next section)

**6b. [Detection2]**

Are study characteristics sufficiently different to exclude study?

1. Yes (proceed to Detection3)
2. No (skip to the next section)

**6c. [Detection3]**

Are sample characteristics sufficiently different to exclude study?

1. Yes (proceed to Detection4)
2. No (skip to the next section)

**6d. [Detection4]**

Are construct's definitions and measures sufficiently different to exclude study?

1. Yes (proceed to Detection5)
2. No (skip to the next section)

**6e. [Detection5]**

Are matched study effects sufficiently different to exclude study?

1. Yes (consider the study as duplicate)
2. No (proceed to the next question)

**REFERENCE INFORMATION**

**7a. [First_Author]**

First author's Last name

**7b. [Second_Author]**

Second author's Last name

**7c. [Third_Author]…… Cont**.

Third author's Last name

**8a. [Pub‐Title]**

Publication title for the primary report. If there is more than one report from the same study exists, use the title for the report that provides the effect sizes. If the **effect size** is reported in multiple reports, use the **earliest title**.

**8b. [Pub‐Year]**

Year of publication for the primary report (**four digits**). If there is more than one report from the same study exists, use the date for the report that provides the effect sizes. If the **effect size** is reported in multiple reports, use the **earliest date**.

**8c**. [**Publication**]

Type of publication for the primary report. If there is more than one type of publication, choose the publication that has the **effect size**. If the effect size is reported in more than one publication, first choose the peer‐reviewed publication over another option or select the report that provides the most effective size.

1. Book
2. Book Chapter
3. Journal Article (peer‐reviewed)
4. Journal Article (non‐peer‐reviewed)
5. Dissertations & Theses
6. Government Report (state/local)
7. Government Report (Federal)
8. Agency (nongovernment) Report
9. Technical Report
10. Conference Paper
11. Working Papers
12. Other Sources ________Specify

98. Cannot tell or unclear

99. NA‐Not applicable

**9a. [Funded]**

Do the authors declare funding details?

1. Yes
2. No

98. Cannot tell or unclear

**9b. [Fund_Source]**

What is the funding source? ____________

**10a. [Researcher_Develop**]

Did one or more of the researchers develop the intervention program?

1. Yes
2. No

98. Cannot tell or unclear

**10b. [Researcher_Eval]**

Is the researcher an independent evaluator?

1. Yes
2. No

98. Cannot tell or unclear

**STUDY CONTEXT AND SAMPLE CHARACTERISTICS**

**11. [Country]**

The country in which the study was conducted

1. United States
2. Canada
3. Mexico
4. Australia
5. United Kingdom (Specify……………)
6. Poland
7. South Africa
8. New Zealand
9. Scandinavian Countries (Specify……………)
10. Asian Countries (Specify……………)
11. Other ___________(Specify……………)

98. Cannot tell or unclear

**12a. [State]**

U.S. state in which the study was conducted

1. Alabama
2. Alaska
3. Arizona
4. Add states…….

51. District of Columbia

52. Single state (unspecified)

53. Multiple states (unspecified)

54. Conducted outside the US

98. Cannot tell or unclear

**12b. (Urban‐Rural]**

Where was the study being conducted?

1. Urban
2. Rural

98. Cannot tell

**13. [Program_initiation]**

Who initiated the program?

1. Schools
2. Probation‐ordered
3. Court‐ordered
4. Agency (juvenile institutions or psychiatric facilities
5. Other (Specify……………….)

98. Cannot tell or unclear

**14. [Participation‐type]**

How were the participants recruited into the program?

1. Voluntary
2. Mandatory

98. Cannot tell or unclear

**15. [Study_year]**

What year was the study conducted?

1. Year (four digits) _______

98. Cannot tell or unclear

**16a**. [**Education_setting**]

**(Ages and grade level will be documented based on the countries classification)**

What type of educational setting in which the study was conducted (US equivalent):

1. Preprimary (Kindergarten and below)
2. Primary School (1st to 6th grade)
3. Secondary School (7th to 12th grade)
4. Multiple settings

98. Cannot tell or unclear

**16b. Education setting and age**

1. Preprimary/early childhood (age 3 to 5)
2. Primary School (age 6 to 11)
3. Secondary (age 12 to 19)

***Sample Characteristics***

The target population should consist of parents of students who are enrolled in K – 12th grade.

**17a. [Target_pop‐char1]**

What is the target population?

1. Entire population (no specific groups targeted)
2. Selective population by classes or schools
3. Special population (i.e., court‐ordered or probation ordered due to truancy or other behavioral issues).
4. Other____________
5. Cannot tell or unclear

**17b. [Target_pop‐char2]**

What is the gender composition of the target population?

1. All male

2. All female

3. Both male and female

98. Cannot tell or unclear

**17c. [Target_pop‐char3]**

What is the age composition of the target population? (Include age range, mean, etc. i.e. provided in the study)

Age range (if available)

1. Age 3 to 5
2. Age 6 to 11
3. Age 12 to 19
4. Mean age _____________________
5. Multiple age setting _________________
6. Other_______________

**17d. [Target_pop‐char4]**

What is the socioeconomic status of the target population?

1. Mostly below the poverty line

2. Mostly above the poverty line

3. Evenly distributed

4. Other (specify) ___________________

98. Cannot tell or unclear

**18. [Sample_race_char‐ 1]**

What is the race/ethnicity of the sample?

1. Percentage of American Indian or Alaskan Native
2. Percentage of Asian
3. Percentage Black or African American
4. Percentage of Hispanic or Latino
5. Percentage Native Hawaiian or Other Pacific Islander
6. Percentage White or Caucasian
7. Other………………………

98. Cannot tell or unclear

**INTERVENTION CHARACTERISTICS**

**19. Intervention characteristics**

**19a. [Brief_Name]**

The name or a phrase that describes the intervention: ……………………………………. .

**19b**. Does the intervention have a theoretical basis?

1. Yes, if yes, mention theory used………………………………………………………. .
2. No

**19c**. Any physical or informational materials used in the intervention?

1. Group discussion
2. Counseling
3. Presentations
4. Other……………

**19d. [Type_Curr]**

What type of curriculum was used? (Specify………………….)

**19e. [Who_Inter]**

Who provided the intervention to parents?

1. School teachers
2. School nonteaching staffs
3. Psychologist
4. Social workers
5. Other…………

**19f. [Mode_Delivery]**

The modes of intervention delivered

1. Face‐to‐face
2. Internet
3. Telephone
4. other mechanisms, (specify)…………. .

**19g. [How_Interv]**

How is the intervention provided?

1. Individually
2. Group

**19h. [Length_Interv]**

What was the length of intervention? Indicate in weeks: ________ (Compute is weeks)

**19i. [Personal_Interv]**

If the intervention was planned to be personalized (customized for the group): (Specify…………………………)

**19j. [Modification]**

If the intervention was modified during the course of the study: (Specify…………………………)

**19k. [Type_Interv]**

What was the nature of the Intervention?

1. school‐based intervention
2. community‐based intervention
3. Law enforcement or court‐based intervention
4. Other_____________

**STUDY DESIGN AND METHODS**

Method of group assignment. This item focuses on the initial group assignment technique before the start of treatment.

**Random or near‐random:**

Random assignment at the individual level, intact group (i.e., classrooms) level, and random assignment after individuals have been matched or blocked, or individuals matched based on a cut‐off score. The quasi‐randomized (like placebo, no treatment, waitlist control, treatment‐as‐usual and alternative treatment) procedure is the method of allocation that should create equivalence between groups. The methods of allocation may include matching individuals on certain relevant characteristics, or comparing on one or more pretest measures, or match the group means for one or more pretest measures.

**20a. [Study_Random_Type]**

What type of randomized study design was used? (Specify…………………)

**20b. [Study_Quasi_Type]**

What type of quasi study design was used? (Specify…………………)

**20c. What is the nature of the comparisons for this study?**

1. Single intervention contrasted with single comparison condition
2. Multiple interventions against a single comparison condition
3. Within one group over time
4. Other (specify…………………………. .)

**20d. What type of comparison condition was used? [dropdown menu]**

1. No treatment
2. Treatment‐as‐usual (………………………………)
3. Alternative treatment (………………………….)
4. Waitlist control
5. Other (specify…………………)

**20e. How were treatment and comparison groups formed?**

1. Random allocation
2. Matching (specify the matching method and matching variables)
3. On basis of score on a specific measure (e.g., diagnosis, specify)
4. Self‐selection
5. Other (specify………………………)
6. Unclear

***Matched Variable List***

In the matching and/or statistical controls, please list all the variables used.

**21a. [Match1]**

**21b. [Match2]……. Add more if necessary**

***Program Structure***

**22. [Program_Struc]**

Was the program highly structured, that is, following a set protocol?

1. Yes

2. No

98. Cannot tell

**23. [Prog‐Consist]**

Did the program implementation remain consistent over time?

1. Yes

2. No (If No, explain,……………)

98. Cannot tell

***Attrition***

If the attrition rate is not specifically provided in the study, use the initial sample size and the final sample size to calculate the attrition rate.

**24a. [Attri_rate]**

Attrition rate if provided ________________ **(Skip to 26d)**

**24b. [Initial_samp]**

What was the initial sample size recruited into the study? __________

**24c. [Final_samp]**

What was the final sample size? ___________

**24d. [Adjust_Attri]**

Were there adjustments for attrition?

1. Yes

2. No

98. Cannot tell

**24e. [Diff_Attri]**

Were there adjustments for differential attrition?

1. Yes

2. No

98. Cannot tell

**25. [Baseline‐Diff]**

Were there adjustments for baseline differences?

1. Yes

2. No

98. Cannot tell

***Outcomes***

This section will include the primary outcomes (i.e., Truancy‐school attendance) and Delinquent behavior (i.e., substance abuse, gang activity, and aggression)

**26a. [Num_Prim_Outcome]**

How many primary outcomes were reported in the study? _____

**26b. [Prim_Outcome1]**

What was the **Primary Outcome1** of the study? _______

98. Cannot tell/researcher did not prioritize outcomes

**26c. [intended_Prim_Outcome1]**

Was this initially intended as a primary outcome of the study? ______

1. Yes

2. No (explain)______________________

98. Cannot tell

**26d. [Prim_Outcome2]**

What was the **Primary Outcome2** of the study? _______

98. Cannot tell/researcher did not prioritize outcomes

**26e. [intended_Prim_Outcome2]**

Was this initially intended as a primary outcome of the study? ______

1. Yes

2. No (explain)______________________

98. Cannot tell

**27a. [Prim_Outcome_Data]**

What source was used for primary outcome data?

1. Official data (from the police, school administrator, court, etc.)
2. Parents' report
3. Teacher's report
4. Self‐report surveys
5. Multiple sources
6. Other (specify) (professional observation, assessment, or diagnosis) ______________

**27b. [Prim_Multiple_Source]**

If multiple sources for primary outcomes were used, specify_____________

**27c. [Official_Data_Primary]**

If official data was used for primary outcomes, what specific type(s) of data was used? (Select all that apply)

1. Police contacts

2. Arrests

3. Court records

4. Convictions

5. Other (specify)

98. Cannot tell

99. N/A (official data not used)

**28a. [List_Second_Outcomes]**

Specify the type of secondary outcomes for the study.

**28b. [Num_Second_Outcomes]**

How many secondary outcomes were reported in the study? _____

98. Cannot tell

**28c. [Second_Outcome_Data]**

What source was used for secondary outcome data?

1. Official data (from the police, court, etc.)
2. Parents' report
3. Teacher's or school administrator report
4. Self‐report surveys
5. Multiple sources
6. Other (specify) (professional observation, assessment, or diagnosis)_______________

**28d. [Second_Multiple_Source]**

If multiple sourced for secondary outcomes were used, specify_____________

**28e. [Official_Data_Secondary]**

If official data was used for secondary, what specific type(s) of data was used? (Select all that apply)

1. Police contacts

2. Arrests

3. Court records

4. Convictions

5. Other (specify)

98. Cannot tell

99. N/A (official data not used)

**29a. [Assess_Quality]**

Did the researcher assess the validity of the data collected?

1. Yes

2. No

98. Cannot tell

**29b. [Quality_Concern]**

Did the researcher(s) express any concerns over the quality of the data?

1. Yes

2. No

98. Cannot tell

**29c. [Quality_Explain]**

If the researchers explained concerns, please indicate below: __________________________________________________________________________________________________________________________________________________________________

**30a. [Match‐Initial_Prob]**

Does the evaluation data correspond to the initially stated problem? (i.e., if the problem is truancy, does the evaluation data look at whether truancy decreased?)

1. Yes
2. No

**30b. [Match‐Initial_No]**

If no, explain the discrepancy: ____________________________________________________________________________________________________________________________________________________________________

**EFFECT SIZE AND STATISTICAL SIGNIFICANCE**

**Dependent Measure Descriptors**

***Sample Size***

**31. [Tot_Sample_Size]**

Based on the unit of analysis for this outcome, what is the total sample size in the analysis?

Indicate the number_________

**32. [Tot_Sample_Treat]**

What is the total sample of the treatment group? Indicate the number**__________**

**33. [Tot_Sample_Cont]**

What is the total sample size of the control group/comparison group? Indicate the number _____

**34a. [Attri_Analy]**

Was attrition a problem in the analysis for this outcome?

1. Yes
2. No

**34b. [Attri_Prob]**

If attrition was a problem, provide details (e.g., how many cases were lost and why they were lost).

__________________________________________________________________________________________________________________________________________________________________

***Effect Size Data***

**35. [Raw_Diff_Fav]**

Does the raw effect size difference favor (i.e., shows more success for)?

1. Treatment group

2. Control group

3. Neither or exactly equal

98. Cannot tell (or statistically insignificant report only)

99. Not Applicable

**36. [Stat_Sing_Groups]**

Does the statistical significance test indicate statistically significant differences between the control and treatment groups?

1. Yes

2. No

98. Cannot tell

99. Not Applicable

**37. [Report_Stad_Effect]**

Was a standardized effect size reported?

1. Yes
2. No

**38a. [Effect_Size]**

If yes, what was the effect size? ______

**38b. [Yes_Effect_Size_Page]**

If yes, what page number was the effect size reported? ________

**39a. [No_Data_Avail]**

If no, is the data available to calculate an effect size?

1. Yes
2. No

**39b. [Cal_Effect_Size]**

What types of data can be used to calculate the effect size?

1. Means and standard deviations
2. t‐value or F‐value
3. Chi‐square (df=1)
4. Frequencies or proportions (dichotomous)
5. Frequencies or proportions (polychotomous)
6. Other

**40a. [Control_Validity]**

Did the evaluation control for validity by using multivariate methods (i.e., regression) to assess the impact of the program on the outcome? ______

1. Yes
2. No

**40b. [Control_Validity_Yes]**

If yes, did this analysis find that the intervention reduced the outcome at a statistically significant level (*p*=.05)?

1. Yes
2. No

***Means, Standard Deviations, Standard Errors, Risk of Bias (actual values needed)***

**41a. [Treat_Group_Mean_prim1]**

What is the treatment group mean for primary outcome1?

**41b. (Control_Group_Mean_prim1]**

What is the control/comparison group mean for primary outcome1?

**41c. [Treat_Group_Mean_prim2]**

What is the treatment group mean for primary outcome2?

**41d. (Control_Group_Mean_prim2]**

What is the control/comparison group mean for primary outcome2?

**42a. [Treat_Group_SD_prim1]**

What is the treatment group standard deviation for primary outcome1?

**42b. [Control_Group_SD_prim1]**

What is the control/comparison group standard deviation for primary outcome1?

**42c. [Treat_Group_SD_prim2]**

What is the treatment group standard deviation for primary outcome2?

**42d. [Control_Group_SD_prim2]**

What is the control/comparison group standard deviation for primary outcome2?

42e‐h. {Standard Error]

***Proportions or frequencies (actual values needed)***

If the primary outcomes 1 and 2 are indicated separately for each group, then the n's and proportions will be reported separately. However, if they are combined in the study, they will be reported under outcome 1.

**43a. [Treat_Group_Outcome1_n]**

What is the “n” of the treatment group with a successful outcome1?

**43b. [Control_Group_Outcome1_n]**

What is the “n” of control/comparison group with a successful outcome1?

**43c. [Treat_Group_Outcome2_n]**

What is the “n” of the treatment group with a successful outcome2?

**43d. [Control_Group_Outcome2_n]**

What is the “n” of control/comparison group with a successful outcome2?

**44a. [Treat_Group_Outcome1_Prop]**

What is the proportion of the treatment group with a successful outcome1?

**44b. [Control_Group_Outcome1_Prop]**

What is the Proportion of a control/comparison group with a successful outcome1?

**44c. [Treat_Group_Outcome2_Prop]**

What is the proportion of the treatment group with a successful outcome2?

**44d. [Control_Group_Outcome2_Prop]**

What is the Proportion of a control/comparison group with a successful outcome2?

***Significance Tests (actual values needed)***

**45a. [t‐value_prim1]**

What is the t‐value for primary outcome1?

**45b**. **[t‐value_prim2]**

What is the t‐value for primary outcome2?

**46a. [F‐Value_prim1]**

What is the F‐value for primary outcome1?

**46b. [F‐Value_prim2]**

What is the F‐value for primary outcome2?

**47a. [Chi‐Square_prim1]**

What is the Chi‐square value (df=1) for primary outcome1?

**47b. [Chi‐Square_prim2]**

What is the Chi‐square value (df=1) for primary outcome2?

***Calculated Effect Size (actual values needed)***

**48a**. [Effect_size]

What is the calculated effect size?

**48b‐48e**. Risk of Bias

**CONCLUSIONS BY THE AUTHOR(S)**

Conclusions about the effectiveness of the intervention in regard to the current outcomes/problem being addressed.

**49. [Conclude_Reduction]**

Did the author(s) conclude that the parent engagement program reduced the problem?

1. Yes

2. No

98. Unclear/no conclusion stated by authors

**50. [Conclude_Benefit]**

Did the author(s) conclude that the parent engagement program was beneficial?

1. Yes

2. No

98. Unclear/no conclusion stated by authors

**51. [Conclude_Relationship]**

Did the author(s) conclude there was a relationship between the parent engagement program and a reduction in delinquency/crime?

1. Yes
2. No
98. Unclear/no conclusion stated by authors

**52. [Conclusion_Notes]**

Additional notes about conclusions:

__________________________________________________________________________________________________________________________________________________________________

**53. [Miss_Data]**

How did the author(s) manage their evaluation of missing data?

1. Listwise deletion
2. Pairwise deletion
3. Mean or mode imputation
4. Single regression imputation
5. Dummy variable approach (imputed value at zero with dummy variable)
6. Multiple imputations
7. Full information maximum likelihood (FIML)
8. Other methods (Specify____________)
98. Cannot tell
99. Not applicable – no missing data

**Sources used to construct the codebook:**

Fay, S., & Eggins, E. (2019). PROTOCOL: Family treatment drug courts for improving parental legal and psychosocial outcomes. *Campbell Systematic Reviews*, *15*(1–2). https://doi.org/10.1002/cl2.1024

Piquero, A. R., Farrington, D. P., Welsh, B. C., Tremblay, R., & Jennings, W. G. (2007). *Effects of early family/parent training programs on antisocial behavior & delinquency*. Available at: https://campbellcollaboration.org/images/pdf/plain-language/Piquero_EFPT_review_1.pdf

Kettrey, H. H., Marx, R. A., & Tanner‐Smith, E. E. (2019). *Effects of bystander programs on the prevention of sexual assault among adolescents and college students*. Available at: https://campbellcollaboration.org/library/bystander-programs-sexual-assault-adolescents-college-students.html
